# On the Broadening of Single-Layer Metasurface Bandwidth by Coupling Resonances

**DOI:** 10.3390/ma13092063

**Published:** 2020-04-29

**Authors:** Humberto Fernández Álvarez, María Elena de Cos Gómez, Fernando Las-Heras Andrés

**Affiliations:** Área de Teoría de la Señal y Comunicaciones, Dpt. Ingeniería Eléctrica, Universidad de Oviedo, Edificio Polivalente, Mod. 8, 33203 Gijón (Asturias), Spain; medecos@uniovi.es (M.E.d.C.G.); flasheras@uniovi.es (F.L.-H.A.)

**Keywords:** metasurface, metasurface absorber, bandwidth broadening, overlapping resonances

## Abstract

In this contribution a new technique to increase the bandwidth of metasurfaces without increasing their profile is presented. This work takes advantage of the potential multiresonant behavior of a metamaterial whose unit cells comprise nested metallization geometries in the same layer. The novelty stems from the possibility of overlapping these resonances for increasing the bandwidth (instead of obtaining a multiresonant metasurface). Several guidelines to achieve the aforementioned bandwidth broadening, which are applicable to all metasurface designs, will be provided. An equivalent circuit model will be used to better explain the presented technique; then, it will be applied to several metasurface absorbers (MTAs), showing not only a bandwidth broadening but also an absorption reinforcement. Measurements will be also presented to corroborate the simulation results.

## 1. Introduction

During recent years, many authors have focused their research on metasurfaces, which are two-dimensional periodic artificial materials that exhibit properties not found in natural ones. Due to such properties, they have been used in many applications, such as cloaking or concealing objects [1], energy harvesting [2], superlenses in optics [3] and electromagnetic imaging [4]. However, it should be mentioned that they exhibit several limitations, such as challenging characterization [5,6], limited angular stability and bandwidth [7,8,9], lack of conformability and manufacturing restrictions [10]. As such, many authors have devoted their efforts to overcome some of the aforementioned limitations.

Metasurfaces have also been designed to absorb or cancel incoming electromagnetic waves. They are two-dimensional periodic metamaterials, whose fabrication complexity is often reduced. Metasurface absorbers (MTAs) are designed to match the surface impedance of the hosting medium and absorb the incoming electromagnetic wave, whilst the checkerboard combination of either perfect electric conductors (PECs) and artificial magnetic conductors (AMCs) or two different AMCs are employed to cancel the incoming electromagnetic wave [11,12].

This paper will be focused on designing MTAs with improved properties. MTAs usually comprise a grid, which is a metallo-dielectric frequency selective surface (FSS), on a grounded dielectric. MTAs, like any metasurface, exhibit limited bandwidth due to their resonant behavior. Many works have been focused on increasing their bandwidth by using thick dielectrics, scaling their unit cell’s metallization geometries in one or more layers [13,14], introducing resistors on their unit cell’s metallizations [15] or employing magnetic superstrates [16]. However, most of these techniques give rise to large profiles.

Other types of resonant absorbers have been also developed. The first of these absorbers was the Salisbury screen (SS), which cancels the wave reflection by resorting to a destructive interference. The Jaumann absorber, based on the same principle as the S.S., achieves a widened bandwidth by adding multiple layers. Following these, the circuit analog (CA) absorber was developed, which consists of a lossy FSS (behaving as an RLC circuit) on a grounded dielectric [17], instead of a resistive layer, as in the case of the Dällenbach absorber. 

The capacitive circuit absorbers (CCAs) are similar to the CA absorbers, but replace the RLC circuit with an RC one. Although the CCAs offer slight reductions in dielectric thickness compared to the aforementioned absorbers, the structure thickness resulting from the application of the mentioned techniques to broaden the bandwidths of metasurfaces is still large.

Consequently, a new technique to improve the bandwidth and reinforce the absorption of MTAs without increasing their profiles would be of major interest. This work pursues this aim, introducing a new technique based on properly designing each metasurface unit cell so that its profile can be kept whilst its bandwidth is broadened. 

The proposed method to overcome the bandwidth limitations of metasurfaces consists is that of overlapping multiple resonances of the presented MTAs. Indeed, in contrast to other authors who achieved multiband operation by nesting scaled versions of the unit cell’s metallization geometry [18], a new technique to overlap these multiple resonances will be provided in this paper. This technique can be achieved by following some guidelines that will be discussed in this contribution.

## 2. Materials and Methods 

**Figure 1:**
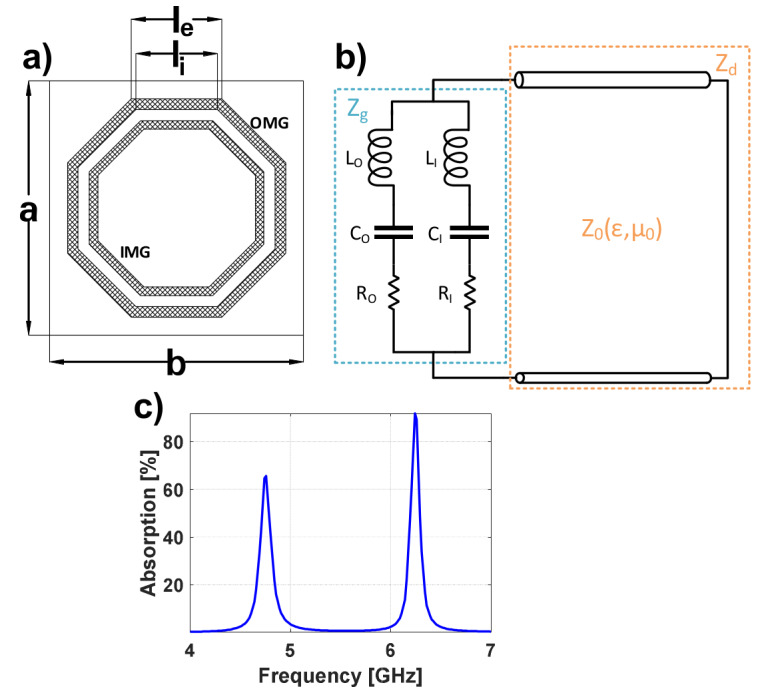
(**a**) Unit-cell geometry of the initial nested metasurface (DS1); (**b**) equivalent circuit model; (**c**) absorption results of the DS1.

Prior to properly designing the unit cell, it is crucial to understand the behavior of an MTA comprising nested unit cells. Therefore, an MTA with canonical unit cells, such as the one presented in Figure 1a, will be firstly analyzed. This unit cell consists of outer and inner metallization geometries (OMG and IMG, respectively) on a commercially available Arlon25N dielectric with $\epsilon_{r}=3.38,\;tan\delta =0.0025$ and 0.762 mm thickness. The OMG has the following parameters: $l_{e}=5.1\;\mathrm{mm},l_{i}=4.6\;\mathrm{mm}$ and a=b=14.33 mm, whereas the IMG is obtained by scaling the OMG by 0.8. This first MTA will be regarded to as DS1.

The equivalent circuit of this type of metasurface can be generically modeled using the equivalent circuit of Figure 1b [19]. The OMG and IMG can be represented as RLC circuits. The transmission line is used to represent the grounded dielectric. Without loss of generality, it will be assumed that $L_{1},\;C_{1}$ and $R_{1}$ are the circuit components that model the OMG and that $L_{2},\;C_{2}$ and $R_{2}$ are the circuit components that model the IMG. 

Assuming that both structures can be decoupled, their resonance frequencies can be computed as follows:

(1) $$f_{r}=\frac{1}{\sqrt{L_{i}C_{i}}}$$

where *I = O* or *I*, depending whether the outer or inner metallization, respectively, is considered. For the case under analysis, one can consider $L_{I}\ll L_{O}$, since the inductance is proportional to the length of the metallization [20,21]. Therefore, the first absorption peak at 4.758 GHz in Figure 1c can be attributed to the resonance of the outer metallization, and the peak at 6.242 GHz can be attributed to the inner metallization [22].

**Figure 2:**
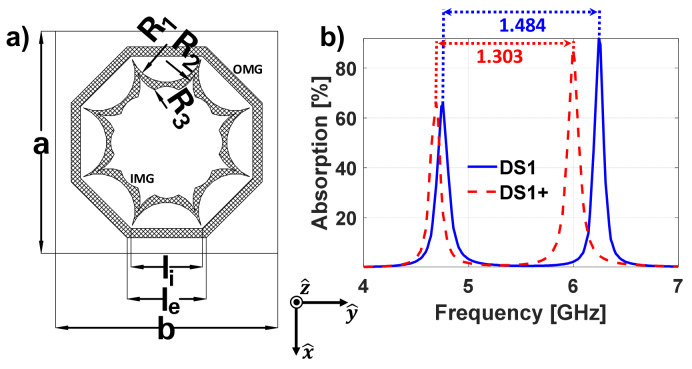
(**a**) Unit cell geometry of the improved nested metasurface (DS1+); (**b**) absorption results of the DS1 and DS1+.

From the previous result, one can clearly notice that the inductance of the inner metallization should be increased, so that both resonances can be brought close to each other. The latter cannot be achieved by using a scaled version of the outer metallization, since it will always exhibit a smaller inductance. Consequently, another inner metallization geometry has to be devised, such as the one presented in Figure 2a, which has the following parameters: $R_{1}=2.4\;\mathrm{mm}$, $R_{2}=2\;\mathrm{mm}$,$\;R_{3}=1.4\;\mathrm{mm}$, $l_{e}=5.1\;\mathrm{mm}$, $l_{i}=4.6\;\mathrm{mm}$ and a=b=14.33 mm. It should be mentioned that the center position of the circumferences of radii $R_{1}$, $R_{2}$ and $R_{3}$ are located in the negative x-axis, considering the coordinate system at the unit cell center, at −b/2, −b_2/2 and −t, respectively, where $b_{2}=4p_{2}+4t_{2}$, $p_{2}=R_{2}sin\left(\alpha \right)$, $t_{2}=R_{2}cos\left(\alpha \right)$, $t=R_{1}cos\left(\alpha \right)$ and $\alpha =22.5^{\circ }$. This MTA will be named DS1+.

From the results (see Figure 2b) it is possible to observe that even though the distance between the absorption peaks has been reduced by more than 100 MHz, it is still wide. Several parametric analyses were conducted trying to bring both resonances closer, but none of them provide the desired resonance overlapping. Therefore, it can be concluded that the equivalent circuit of either the inner or outer metallization geometries should be modified, so that the frequency positions at which the resonances arise can be reversed. The latter means that the resonance attributable to the OMG should appear at higher frequencies than that of the IMG, or vice versa. Then, by scaling one or the other metallization geometry, the resonances could be overlapped.

## 3. Results and Validation of the Method

**Figure 3:**
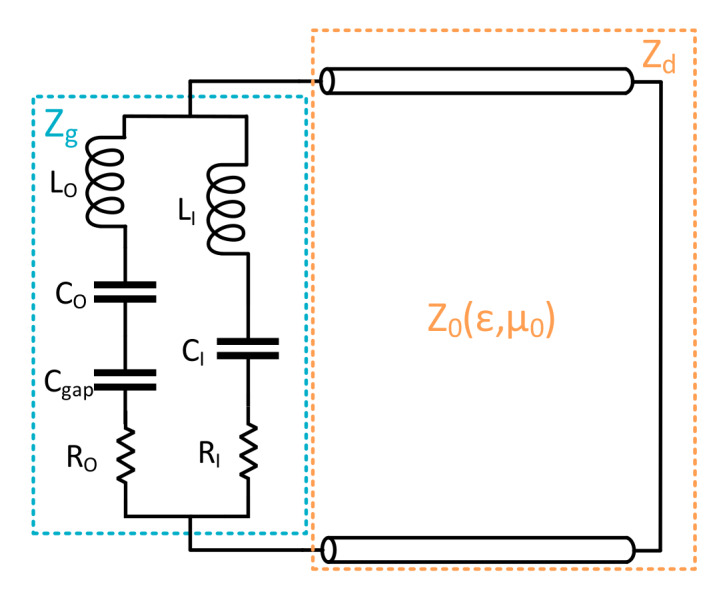
Equivalent circuit model of the new proposed metasurface.

In this paper, it is chosen to modify the outer metallization, so that its equivalent circuit model can be altered. For achieving this goal, a gap is introduced in the outer metallization, so that a capacitance $C_{gap}$ is added (see Figure 3). After performing this modification, it was observed that the resonance attributable to the outer metallization will appear at higher frequencies than the inner one.

**Figure 4:**
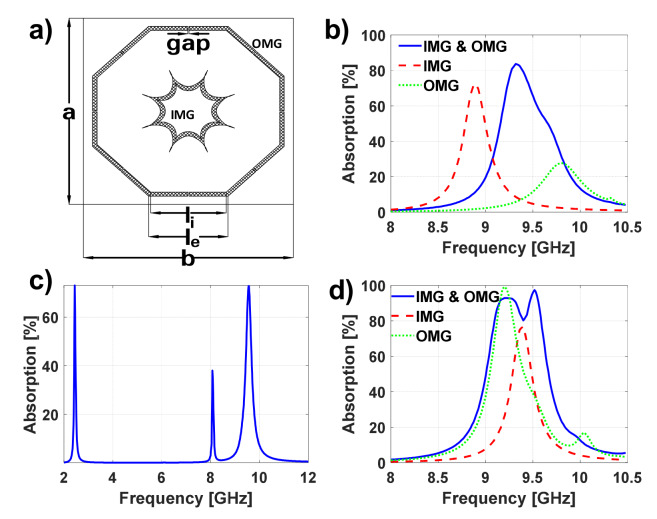
(**a**) Unit cell geometry of the first proposed metasurface absorber (MTA) with overlapping resonances. (**b**,**d**) Absorption results of the DO1 and the MTA whose grids consist of the IMG or OMG using (**b**) Arlon 25N as dielectric and (**d**) FR4. (**c**) Absorption results of the DO1 when gap is removed.

The first proposed metasurface is presented in Figure 4a. Initially, the OMG and the IMG are designed independently to resonate around 9 GHz (an interesting frequency for radar applications) using the same grounded dielectric (Arlon25N of 1.524 mm thickness). After several parametric adjustments aimed at overlapping the resonances, it was found that the outer metallization should have the following parameters: $l_{e}=8.68\;\mathrm{mm}$, $l_{i}=8.23\;\mathrm{mm}$, gap=0.6 mm and a=b=21.97 mm. It can be noticed that the inner metallization was slightly modified with respect to the one in Figure 2a. It is now composed of two intersected ellipses (instead of circles), having semi-major axes of 4 and 3.5 mm and semi-minor axes of 2 and 1.75 mm, with center positions on the x-axis at 6.27 and 5.23 mm, respectively. This metallization is obtained by intersecting these ellipses and duplicating the resultant structure each $45^{\circ }$. Then, a scaling factor of 0.98 is applied to the inner metallization, so that both resonances overlap, as seen in Figure 4b. In this figure, the absorption of the MTA considering both the IMG and OMG (in blue), which will be called DO1, and the absorptions obtained when considering just the IMG or OMG (in red and green, respectively) are presented.

From the results, it can be clearly noticed that not only is the bandwidth broadened, but also the absorption is reinforced (84%). 

**Figure 5:**
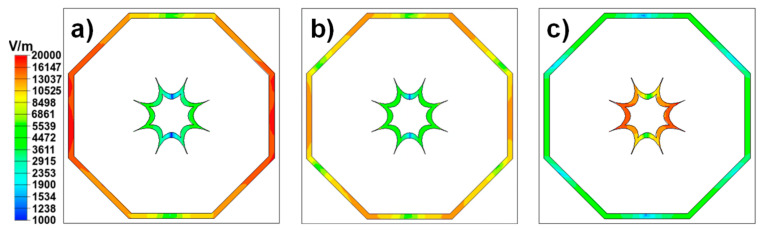
Electric fields of the DO1 when the gap is removed, at the absorption peaks of (**a**) 2.439, (**b**) 8.077 and (**c**) 9.553 GHz.

Figure 4c shows the absorption properties of the structure when the gap is removed. One can notice that the fundamental resonance of the OMG shifts downwards to 2.439 GHz. The peak that appears around 8.077 GHz is due to the excitation of the OMG second mode, as one can observe in Figure 5, where the electric fields on the IMG and OMG are shown. It should be noticed that this second mode is weaker than the fundamental one (providing a much smaller absorption peak).

As a perfect absorption is not obtained, several modifications can be introduced on the MTA to achieve it, such as the adjustment of the grid geometric parameters, the introduction of resistors or the modification of the dielectric. 

In this case, an FR4 dielectric of 0.8 mm will be now considered, and a scaling factor of 0.81 is applied to the IMG to couple both resonances. The results are depicted in Figure 4d. Moreover, in Table 1 the results depicted in Figure 4b,d are detailed, and the resonance frequency ($f_{r}$), absorption peak ($A_{p}$), bandwidth at 50% of absorption ($BW_{50\%})$ and the full width at half maximum (FWHM) are presented. Consequently, one can clearly quantify not only the improvement in the bandwidth, but also the reinforcement of the absorption peak when the resonances attributable to the IMG and OMG are coupled. It should be mentioned that the $BW_{50\%}$, which is a more interesting parameter for assessing the performance of nonperfect absorbers, is almost doubled in both cases.

**Figure 6:**
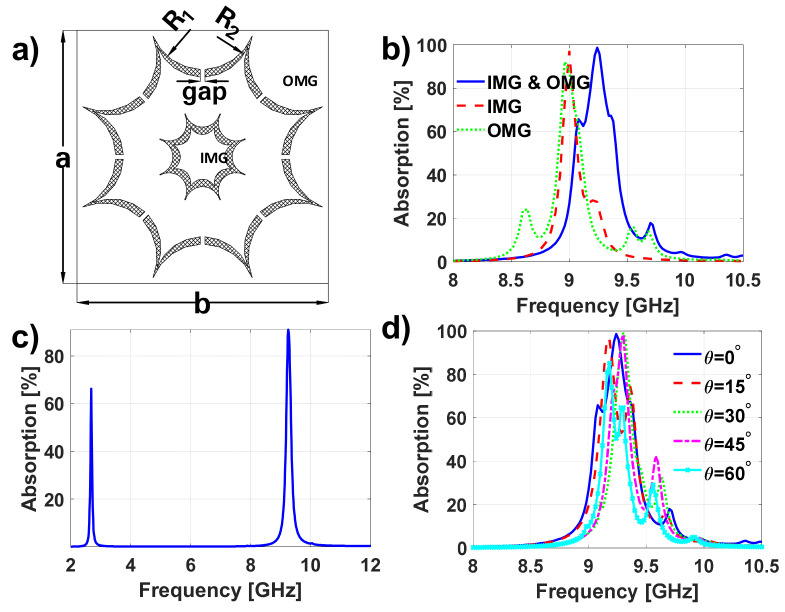
(**a**) Unit cell geometry of the second proposed MTA with overlapping resonances (DO2) and (**b**) the absorption results of the DO2 and the MTA whose grids consist of the IMG or OMG. (**c**) Absorption results of the DO2 when the gap is removed. (d) Angular stability analysis of the DO2.

Aiming at further validating the proposed method, it is applied to another designed MTA. The new structure is the one presented in Figure 6 (DO2). It has the following geometric parameters: $R_{1}=4.65\;\mathrm{mm}$, $R_{2}=4.25\;\mathrm{mm}$, gap=0.4 mm and a=b=21.74 mm. The center position of the circumferences to achieve the OMG can be obtained as in the previous section for the IMG of DS1, by using the new values of $R_{1}$ and $R_{2}$. For better manufacturing tolerance, the IMG is intersected with a circumference of radius 3.6 mm and scaled by a factor of 1.11. In this case, aiming at obtaining a low profile, Arlon25N with a thickness of just 0.762 mm is considered.

The results from the application of the proposed method (introducing gaps in the OMG to change its equivalent circuit model) are presented in Figure 6b. One can clearly notice not only a bandwidth broadening, but also an absorption reinforcement. Figure 6c shows the behavior of the metasurface when the gaps on the OMG are removed. It can be observed that the resonances attributable to the IMG and OMG appear far away from each other.

On the other hand, the structure is analyzed under different incidence angles showing proper absorption and bandwidth behavior for a range of incidence angles from 0 to 60°.

One should notice that the angular sensitivity is strongly dependent on the dielectric properties of the substrate, which are not modified in the application of this technique; hence, the angular sensitivity of the final metasurface will highly depend on the initial one.

In Table 2, the results obtained for this new proposed metasurface are presented. Once again, it is possible to corroborate not only a bandwidth widening (the bandwidth is almost doubled), but also an absorption reinforcement. Therefore, the proposed method is once more proved.

**Figure 7:**
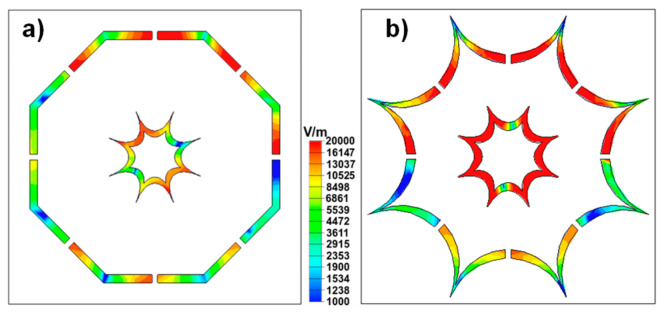
Electric fields of the DO1 when the gap is removed at the absorption peaks of (**a**) 2.439, **(b**) 8.077 (**c**) and 9.553 GHz**.**

To further clarify the previous ideas, the electric fields at the resonance frequencies of the proposed MTAs are analyzed. The electric field of the DO1 on FR4 at 9.22 GHz and the DO2 at 9.24 GHz are presented in Figure 7a and Figure 7b, respectively. It can be observed that both metallic structures are excited at the absorption peaks. Therefore, the resonance overlapping phenomena is once more verified.

Finally, the presented method will be compared with others already in the literature. It should be noticed that the metamaterial absorber bandwidth depends on the operation frequency band, the dielectric properties and the unit cell geometry. For a given relative permittivity and loss tangent, increasing the dielectric thickness broadens the bandwidth. On the other hand, it is easier to obtain wider absolute bandwidth at higher frequencies. 

Moreover, one should keep in mind that the aim of the paper is not to show the best possible design in terms of bandwidth, but rather to introduce a novel method that broadens the bandwidth of a given metasurface absorber and exhibits certain advantages, namely that it requires no profile modification, exhibits symmetry preservation, is easy to be implement and is frequency-scalable, among other advantages.

For a fair comparison and given that the final achievable MTA bandwidth is dependent on the initial one, the different methods in the literature will be compared in terms of bandwidth improvement from the initial MTA to the final one after applying each method. 

Apart from increasing the dielectric thickness to increase the bandwidth (which is a well-known method), other methods have been presented in the literature and applied to different metasurface absorber unit cells [23]. As previously mentioned, these methods are the scaling of the unit cell’s metallization geometries in the same layer (horizontal scaling [24,25]) or in stacked layers (vertical scaling [26], the design of fractal structures [27], the employment of magnetic materials [28] or the introduction of lumped resistors [29].

For an optimum comparison, the compared structures should be in the same frequency band. However, most authors do not provide the required data for such comparison.

The following table presents different works in which the aforementioned methods were applied and compares them with the one proposed in this paper:

Although it seems that the method based on introducing lumped resistors improves the metasurface absorber bandwidths more, it is a different technique from the others as it is based on introducing lumped components, which increases the fabrication cost and greatly increase the profile (due to the resistor height and the circuit analog absorber consideration on which most of the works are based [30]). The manufacturing process is also arduous, as these resistors have to be precisely welded. On the other hand, this technique does not always ensure a broadening in the bandwidth [31], as the unit cell geometry has to be properly designed and the dielectric must be properly chosen, and may also fully shift the frequency band from the initial structure [29].

It should also be mentioned that this technique is only suitable at microwave frequencies, since at higher ones the resistor parasitic effects increases, its availability is reduced and a more precise mounting procedure is required.

One of the major drawback of applying the horizontal scaling method, apart from increasing the unit cell size, is the difficulty in retaining the symmetry of the unit cells. As different scaling factors are applied to the initial unit cell metallization geometry, the resulting unit cell is not symmetric, which gives rise to worse angular stability. Moreover, slightly lower bandwidth improvement is obtained as compared to the proposed method, as one can see from the previous table. 

On the other hand, the vertical scaling is an alternative that clearly increases the metasurface thickness. Moreover, it can give rise to manufacturing difficulties, as misalignments between the layers may occur when they are arranged.

Complex structures have also been introduced to increase the bandwidth, as is the case of the fractal structures presented in [27]. However, though the symmetry can be kept, it is not easy to completely couple the resonances; hence, absorption valleys may appear between them [27]. Moreover, there are no guidelines for designing this type of structure.

The use of magnetic materials, though it improves the metasurface bandwidth, may increase the metasurface weight and complicate its manufacturing. Moreover, such materials are not as commonly available as the dielectric ones.

Consequently, it could be mentioned that the proposed technique, which considers the worst case (broadest initial bandwidth of IMG or OMG), provides proper bandwidth improvement, without increasing the metasurface profile or degrading the unit cell symmetry, and it can be applied to any frequency band of interest. Moreover, it is easily applicable to any metasurface, following the guidelines provided in the paper. 

Due to the versatility of the method, it can be combined with the other ones presented in Table 3 to further broaden the bandwidth [32,33].

## 4. Experimental Validation and Discussion

**Figure 8:**
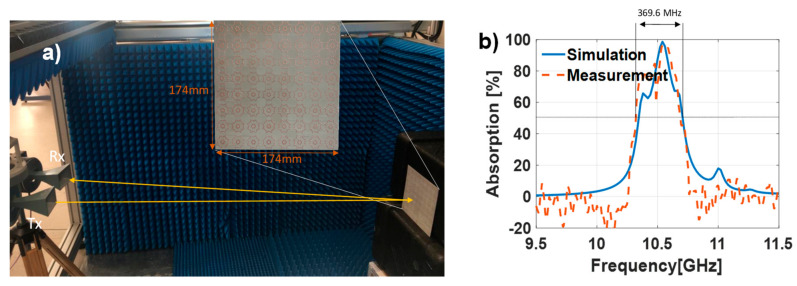
(**a**) Manufactured prototype (DO2) and semi-anechoic measurement set-up. (**b**) Measured vs. simulation absorption results of the DO2.

Due to the current availability of Arlon 25N with 0.762 mm thickness in our facilities, the DO2 presented in Figure 6 was manufactured, using a PCB prototyping plotter LPKF ProtoMat H100. The manufactured prototype is presented in Figure 8a and has 8 × 8 unit cells with a total dimension of 174×174 mm. The measurements were conducted in a semi-anechoic environment and under normal incidence using two Narda 640 waveguide horn antennas, one acting as transmitter and the other as receiver. The set-up calibration was conducted using an identical-sized metallic plate as the MTA. To conduct proper measurements under far-field condition in a frequency band from 8−12 GHz, the distance between the MTA and the prototype should be about 4.8 m [34]. This distance may be excessive for some measurement set-ups, even in an anechoic chamber. However, as shown in [35], proper measurements with small errors can be obtained when measuring the prototype under normal incidence in the Fresnel region. 

Owing to the manufacturing tolerances and deviations in the dielectric properties and thickness, there is a slight shift in the resonant frequency of the measured prototype. For a clearer comparison in terms of absorption and bandwidth, the simulation results are superposed with the measurement ones (see Figure 8b). From the measurement results, one can observe suitable absorption around 10.5 GHz, with a 3.52% relative bandwidth at 50% of absorption. The slight frequency shift compared to simulation can be attributed to manufacturing tolerances and deviations in the dielectric properties and thickness. Nevertheless, one can also notice the enlargement of the absorption bandwidth due to the coupling between the resonances attributable to the IMG and OMG.

Until now, other authors have achieved multiband operation by nesting scaled versions of the unit cell’s metallization geometry [17]. Other studies have been devoted to broadening the metasurface bandwidth at the expense of increasing its profile. However, no study has been addressed to increasing this bandwidth without increasing the metasurface profile [12,13,14,15]. Consequently, this paper has tackled this concern and shown that the metasurface bandwidth broadening can be attained by properly designing the unit cell’s metallization geometries so that their resonance frequencies can be overlapped. Moreover, the presented technique is not restrictive; other methods, such as the increase of the dielectric thickness, the introduction of magnetic superstrates or resistors or the implementation of the capacitive circuit method, can be additionally applied to the considered metasurface to further increase its bandwidth.

## 5. Conclusions

In this paper, a new technique to increase the bandwidth of metasurface absorbers (MTAs) comprising two independent nested metallization geometries in the same unit cell has been presented. It is shown for the first time that by properly devising the aforementioned metallization geometries, instead of obtaining a multiresonant MTA, a broadband MTA can be developed, since their resonances can be overlapped. Indeed, the key aspect in obtaining this overlapping was found to be the modification of the equivalent circuit of either the inner or outer metallization geometry so that their resonance frequencies can be swapped. Hence, the resonance frequency attributable to the inner metallization appears at a lower frequency than the that of the outer metallization. As an additional reward, the absorption peak when both resonances overlap has been reinforced. Good agreement between simulation and measurements has been also obtained in terms of coupling the resonances and reinforcing the absorption.

In this first analysis, only two metallization geometries have been considered, but this technique can be applied to any metasurface and to cases where more than two metallization geometries are nested.

## Figures and Tables

**Table 1:** Resonance frequency, absorption peak and bandwidth of the DO1 when considering the full metallization or just the IMG or OMG for different dielectric substrates.

		$f_{r}$ (GHz)	$A_{p}$ (%)	$BW_{50\%}$ (%)	FWHM (%)
Arlon25N 1.524 mm	**IMG and OMG**	**9.32**	**84**	**5.38**	**6.51**
	IMG	8.88	73	2.23	3.42
	OMG	9.81	28	0	5.38
FR4 0.8 mm	**IMG and OMG**	**9.52**	**98**	**6.93**	**7.07**
	IMG	9.4	77	2.25	2.95
	OMG	9.2	99	4.02	4.02

**Table 2:** Resonance frequency, absorption peak and bandwidth of the DO1 when considering the full metallization or just the IMG or OMG.

		$f_{r}$ (GHz)	$A_{p}$ (%)	$BW_{50\%}$ (%)	FWHM (%)
Arlon25N 0.762 mm	**IMG and OMG**	**9.24**	**99**	**3.92**	**3.92**
	IMG	9	97	1.56	1.56
	OMG	8.96	92	2.36	2.36

**Table 3:** Comparison of the method with others previously presented in the literature.

Ref.	Method	$f_{c}$ *(GHz)*	Thickness (mm)*	Dielectric	*Initial BW* *(%)*	*Final BW* *(%)*	*BW* Improvement *(times)*	Symmetry Keeping
[26]	Horizontal Scaling	10.37	1.07 $\lambda_{g}/12.5$	FR4 ($\varepsilon_{r}=4.4)$	3.86%	6.56%	1.7	No
[27]	Horizontal Scaling	5.26	1.67 $\lambda_{g}/15.4$	FR4 ($\varepsilon_{r}=4.4)$	7.79%	10.91%	1.4	No
[28]	Vertical Scaling	4.91	0.97 $\lambda_{g}/28.9$	FR4 ($\varepsilon_{r}=4.4)$	3.2%	7.91%	2.5	Yes
[29]	Fractal Structure	9.32	1.67 $\lambda_{g}/8.4$	FR4 ($\varepsilon_{r}=4.4)$	10.73%	18.56%	1.7	Yes
[30]	Magnetic material	2.65	2.42 $\lambda_{g}/15.9$	-	41.5%	76.63%	1.9	Yes
[31]	Lumped Resistors	4.1	5.034^**^ $\lambda_{g}$/5.15	FR4 ($\varepsilon_{r}=4.4)$	4.94%	68.8%	13.9	Yes
This Work (DO1)	Nested Coupling	9.52	0.836 $\lambda_{g}$/17.4	FR4 ($\varepsilon_{r}=4.4)$	4.02%	7.07%	1.8	Yes
This Work (DO2)	Nested Coupling	9.24	0.798 $\lambda_{g}$/21.7	Arlon25N ($\varepsilon_{r}=3.38)$	2.36%	3.92%	1.7	Yes

$\lambda_{g}$ is the guided wavelength at the highest operation frequency of the structure. ^**^ Resistor height not considered.
